# Heterogeneity of Host TLR2 Stimulation by *Staphylocoocus aureus* Isolates

**DOI:** 10.1371/journal.pone.0096416

**Published:** 2014-05-08

**Authors:** Dina Hilmi, Marijo Parcina, Daniel Stollewerk, Jenny Ostrop, Michaele Josten, Alina Meilaender, Ulrich Zaehringer, Thomas A. Wichelhaus, Gabriele Bierbaum, Klaus Heeg, Christiane Wolz, Isabelle Bekeredjian-Ding

**Affiliations:** 1 Department for Infectious Disease, Medical Microbiology and Hygiene, University Hospital Heidelberg, Heidelberg, Germany; 2 Institute for Medical Microbiology, Immunology and Parasitology, University Hospital Bonn, Bonn, Germany; 3 LG Immunchemie, Research Center Borstel, Borstel, Germany; 4 Institute of Medical Microbiology and Infection Control, Hospital of Goethe-University, Frankfurt am Main, Germany; 5 IMIT-Interfakultäres Institut für Mikrobiologie und Infektionsmedizin, Universitätsklinikum Tuebingen, Tuebingen, Germany; Duke University Medical Center, United States of America

## Abstract

High lipoprotein expression and potent activation of host Toll-like receptor-2 (TLR2) are characteristic features of the staphylococcal species. Expression of TLR2 in the host is important for clearance of *Staphylococcus aureus* infection and host survival. Thus, we hypothesized that bacterial regulation of its intrinsic TLR2-stimulatory capacity could represent a means for immune evasion or host adaptation. We, therefore, compared clinical *S. aureus* isolates in regards to their TLR2 activation potential and assessed the bacterial factors that modulate TLR2-mediated recognition. *S. aureus* isolates displayed considerable variability in TLR2-activity with low to absent TLR2-activity in 64% of the isolates tested (68/106). Notably, strain-specific TLR2-activity was independent of the strain origin, e.g. no differences were found between strains isolated from respiratory specimen from cystic fibrosis patients or those isolated from invasive disease specimen. TLR2-activity correlated with protein A expression but not with the *agr* status. Capsule expression and small colony variant formation had a negative impact on TLR2-activity but any disruption of cell wall integrity enhanced TLR2 activation. Altogether, heterogeneity in host TLR2-activity reflects differences in metabolic activity and cell wall synthesis and/or remodeling.

## Introduction

Toll-like receptors (TLR) are crucial for the sensing of invading pathogens and the initiation of early innate immune responses against these. For many years TLRs have been viewed as pattern recognition receptors (PRR) that promote pro-inflammatory cytokine responses and prime Th1 and Th17 responses. To date, however, it is being more and more appreciated that TLRs also possess anti-inflammatory properties and thereby contribute to the limitation and the resolution of the immune response.

TLR2 is one of the most ubiquitously expressed TLRs: it is found in nearly all immune cells as well as in cells of epithelial and endothelial origin. It forms homo- and heterodimers with TLR1 and TLR6 [1]. Together these receptors form pockets that incorporate mature bacterial lipoproteins depending on their acylation status [2], [3]. Albeit lipoproteins are found in nearly all bacterial species that possess a cell wall, recognition via TLR2 is mainly of interest in Gram positive organisms as these cannot be sensed based on lipopolysaccharide-mediated activation of TLR4. Interestingly, high TLR2-activity has been described as a characteristic feature of the staphylococcal species and several studies have highlighted the importance of TLR2 in immune defense against *S. aureus* [4], [5].

Genes encoding staphylococcal lipoproteins such as SitC are found among the core genes, but high numbers of lipoproteins are also found as accessory elements encoded in the pathogenicity island vSAα of *S. aureus* [6], [7]. Next to enabling TLR2 recognition the main function attributed to mature lipoproteins is iron uptake and storage, which represents a growth advantage for staphylococci [8], [9]. As a consequence, lack of mature lipoprotein expression reduces the pro-inflammatory immune response, but also decreases intracellular survival of the bacterium [8], [10].

During chronic persistent infection and colonization bacteria display less metabolic activity than during invasion. In views of the dual role of lipoproteins in bacterial metabolism and immune recognition we reasoned that bacterial control of TLR2-activity might be associated with immune evasion or host adaptation. To investigate this hypothesis, we analyzed TLR2-activity in clinical isolates and determined the bacterial factors influencing TLR2-activity.

## Materials and Methods

### Bacterial Strains and Culture

*S. aureus* clinical isolates were isolated from clinical specimen referred to the routine medical microbiology laboratory of the Department of Infectious Diseases, University Hospital Heidelberg, Germany. Clinical isolates were numbered according to their origin, e.g. isolates cultured from patients with invasive diseases were categorized as “INV” and those obtained from respiratory specimen of cystic fibrosis patients as “CF”. Storage of the bacterial strains at −80°C was performed in skim milk (Becton Dickinson, Heidelberg, Germany) or with cryobeads (Cryobank™, Mast Diagnostica, Reinfeld, Germany). Reference and mutant *S. aureus* strains are listed in Table 1. For each experiment, bacteria were prepared, thawed and cultured freshly on Columbia/5% sheep red blood agar plates (Becton Dickinson, Heidelberg, Germany), Mueller Hinton agar plates (BioMerieux, Nuertingen, Germany), or CASO-bouillon (Becton Dickinson, Heidelberg, Germany) and incubated at 37°C overnight.

**Table 1:** *S. aureus* strains used in this study.

Strain	Source	Inserted resistancecassettes	ATCC number orReferences
**CF-#**	clinical laboratory, Heidelberg, Germany	n.a.	cultured from respiratory specimen ofpatients with cystic fibrosis; this study
**CF30, CF17, CF38**	previously published as SCV1, SCV2 and SCV3	n.a.	Hilmi et al. 2013
**INV-#**	clinical laboratory, Heidelberg, Germany	n.a.	cultured from patients with invasiveinfections; this study
**SA113**	Friedrich Götz, Tuebingen, Germany	n.a.	ATCC #35556
**SA113 ** ***ΔhemB***	Friedrich Götz, Tuebingen, Germany	*ermB*	Gaupp et al. 2010
**SA113 ** ***Δsdh***	Friedrich Götz, Tuebingen, Germany	*Spectino-mycin*	Gaupp et al. 2010
**SA113 ** ***Δlgt***	Friedrich Götz, Tuebingen, Germany	*ermB*	Stoll et al. 2005
**Cowan I (SAC)**	DSMZ, Braunschweig, Germany	n.a.	DSM # 20372
**Wood46**	DSMZ, Braunschweig, Germany	n.a.	DSM #20491
**SH1000**	Thomas Wichelhaus, Frankfurt, Germany	n.a.	Besier et al. 2007
**SH1000 Δ** ***thyA***	Thomas Wichelhaus, Frankfurt, Germany	*ermB*	Besier et al. 2007
**Newman-132**	Christiane Wolz, Tuebingen, Germany	IPTG-inducible *cap5A*	Jansen et al. 2013
**RN4220**	Gabriele Bierbaum, Bonn, Germany	n.a.	ATCC # 35556
**Newman**	Christiane Wolz, Tuebingen, Germany	n.a	Wolz et al. 1996
**Newman ** ***Δagr***	Christiane Wolz, Tuebingen, Germany	*tetM*	Wolz et al. 1996

n.a. = not applicable.

### Preparation of Human Peripheral Blood Leukocytes and Cytokine Measurements

The use of human leukocytes was approved by the ethics committees in Heidelberg (approval #157/2006). Written consent was obtained from all donors. Total PBMC were isolated from healthy adults using Ficoll density gradient centrifugation. 4×10^5^ PBMCs were seeded in 96-well flat bottom plates and non-adherent cells removed after 1.5 hours by washing in pre-warmed PBS. Cells were resuspended in RPMI 1640 supplemented with 10% human autologous serum, 1% penicillin/streptomycin, 1% L-glutamine and 0.01 M HEPES and incubated at 37°C and 5% CO_2_ overnight before stimulation.

Cytokine concentrations were determined in supernatants harvested 24 hours after stimulation using the human Flowcytomix™ Th1/Th2 plex (Bender Medsystems, Vienna, Austria): cytokine bead and biotin mixes were added to the supernatants for 2 hours; streptavidin-PE (Phycoerythrin) was added for one hour; after washing cytokine levels were measured on a FACS Canto I flow cytometer (BD Biosciences, Heidelberg, Germany) and analyzed using the software provided by the manufacturer.

### HEK293 Cell Assay for TLR2-activity

Human embryonic kidney (HEK) 293 cells were purchased at the DSMZ (ACC305), grown and seeded at 5×10^4^ cells/well (200 µl/well) in 96-well flat bottom plates and incubated at 37°C and 5% CO_2_ overnight. On the second day the medium was substituted by 150 µl/well of antibiotic- and serum-free medium (OptiMEM, Invitrogen, Karlsruhe, Germany). HEK293 cells were transiently transfected with a plasmid bearing TLR2 cDNA at a concentration of 100 or 200 ng per well, as indicated in the figure legends [11]. Transfection was performed using the cationic lipid agent lipofectamine 2000 (Invitrogen) as previously described [12]. The pDNA/lipofectamine mixture was incubated at room temperature for 20 minutes before transfection. Control cells were treated with lipofectamine only as negative control. After 18 hours, cells were washed with pre-warmed PBS and resuspended in 150 µl/well HEK medium (RPMI (Invitrogen) supplemented with 1% penicillin/streptomycin (PAA laboratories, Coelbe, Germany) and with 10% FCS (Invitrogen). In some experiments, the penicillin/streptomycin supplement was substituted by other antibiotics at concentrations found to lie above the minimal inhibitory concentration of the strains used in the experiments (i.e. SA113), e.g. penicillin (100 IE/ml), vancomycin (150 µg/ml), fosfomycin (25 µg/ml), ciprofloxacin (10 µg/ml) (all from Sigma-Aldrich, Steinheim, Germany) and linezolid (4 µg/ml) (Pfizer, Karlsruhe, Germany).

Stimulation of TLR2-transfected HEK293 cells was performed with purified SitC (provided by F. Götz, Tübingen), Pam_3_CSK_4_ as positive control at the indicated concentrations. Unstimulated cells or cells stimulated with lipofectamine only (Lf) were used as negative controls. Stimulation of cells with different bacterial strains was performed with an MOI of 10∶1 (bacteria/cells). This was achieved by preparing a suspension of bacteria in 0.9% saline solution at an optical density of McFarland (McF) = 1 (corresponding to 3×10^8^ CFU/ml) or equivalent density in case of SCV; 50 µl of this suspension were diluted in 700 µl of RPMI 1640 medium (Invitrogen) and 50 µl/well added for stimulation. For crude (“boiled”) preparations bacterial suspensions were standardized by density (McF = 2) and heated for 15 minutes in a water bath at 100°C. 50 µl of this suspension were added to each well. Preparation of heat-inactivated bacteria was carried out at 60°C for 30 minutes, followed by several washing steps; a bacterial suspension with a density of McF = 1 was diluted as described above.

After stimulation, the plate was incubated for 18–20 hours at 37°C and 5% CO_2_; cellular supernatants were harvested and frozen in −20°C until used for human IL-8 ELISA (BD Opteia ELISA kit, BD Biosciences, Heidelberg, Germany).

### Assessment of TLR2-activity upon IPTG-dependent Capsule Expression

For the impact of capsular polysaccharides on host TLR2-activity, pTLR2-transfected HEK293 cells were stimulated with *S. aureus* Newman-132 (cultured in LB medium/2% sodium chloride for 6 hours with or without 1 mM IPTG) in RPMI/10% FCS with no antibiotic supplement. Expression of capsular polysaccharides was verified after staining with anti-cp5 as described in [13]. After 2.5 hours, cells were washed 3× with PBS and resuspended in RPMI/10% FCS supplemented with ciprofloxacin (10 µg/ml). After 18–20 hours at 37°C and 5% CO_2_ cellular supernatants were harvested for IL-8 ELISA as described above.

### Bacterial Protein Lysates and Western Blot Analysis

Bacteria were cultured in CASO-bouillon until they reached an OD_600 nm_ = 1. Cells were washed twice in saline solution, the pellet resuspended in lysis buffer supplemented with protease inhibitors all purchased from (Sigma-Aldrich, Steinheim, Germany) as described in [14]. For proper destruction of the cell wall, the lysates were treated with glass beads (diameter 0.1 mm; Biospec, Bartlesville, OK, USA) and in a bead beater (3×1 minute), centrifuged and the supernatants were kept at 20°C until further use. 10 µg of bacterial proteins were loaded per lane on 12% SDS-PAGE. PageRule™ plus prestained (Fermentas, St. Leon-Rot, Germany) or MagicMark (Invitrogen) were used as protein standards. The proteins were transferred onto nitrocellulose membrane (Schleicher & Schueller, Dassel, Germany) by blotting under semi-dry conditions for 1 hour. The membrane was blocked in TBS/5% skim milk/5% FCS/0.2% Tween20 supplemented with 1% rabbit serum (Sigma-Aldrich) overnight and incubated with mouse monoclonal anti-staphylococcal protein A (Sigma-Aldrich) or with 1% human serum followed by incubation with HRP-conjugated goat-anti-mouse IgG or biotinylated-goat-anti-human IgG (Dianova, Hamburg, Germany) and streptavidin-HRP: 1∶2000 dilution (Millipore).

### Cell Wall and Lipoprotein Preparations

Crude cell wall and lipoprotein fractions of *S. aureus* Cowan strain I (SAC) and Wood46 were prepared as previously described [12]. In brief, cell walls were isolated after mechanical disruption, treated with chloroform/methanol, washed, cooked in 8% SDS. After SDS removal cell walls were treated with 48% hydrofluoric acid (HF), digested with RNAse A (Sigma) and DNAse I (Roche, Mannheim, Germany) and finally with proteinase K (Roche). Enriched lipoproteins were purified from cell walls digested with RNAse and DNAse using 10 mM *N*-octyl-β-D-glucopyranoside (Sigma) and subsequently treated with or without proteinase K (Roche) or lysostaphin (Sigma).

### Assessment of *agr* Activity

Assessment of *agr* activity was based on detection of delta-hemolysin as described in [15]. In brief, isolates to be tested were cross-streaked to the beta-haemolysin-producing *S. aureus* strain RN4220 on Columbia/5% sheep red blood agar. Inhibition of alpha-haemolysin by RN4220 beta-heamolysin allows visualization of delta-haemolysin activity, which is used as an indicator for *agr* activity [16]. Delta toxin expression was further confirmed by MALDI-TOF MS analysis on a Biflex III (Bruker Daltonik GmbH, Bremen, Germany) as described in [17]. In brief, colonies were picked from Columbia sheep red blood agar plates (Becton Dickinson), placed directly on the target, overlaid with matrix solution (saturated L-cyano-4-hydroxycinnamic acid in 50% acetonitrile/2.5% trifluoroacetic acid) and dried. MS spectra were obtained in linear positive mode and data analyzed using the flexAnalysis software (Bruker Daltonik GmbH).

### Statistics

Statistical significance was determined using the paired two-tailed students T-test (*p≤0.05 and **p≤0.005) using Microsoft office Excel version 2003.

## Results

### TLR2-active Lipoproteins Increase Proinflammatory Cytokine Production Induced by *S. aureus*

**Figure 1:**
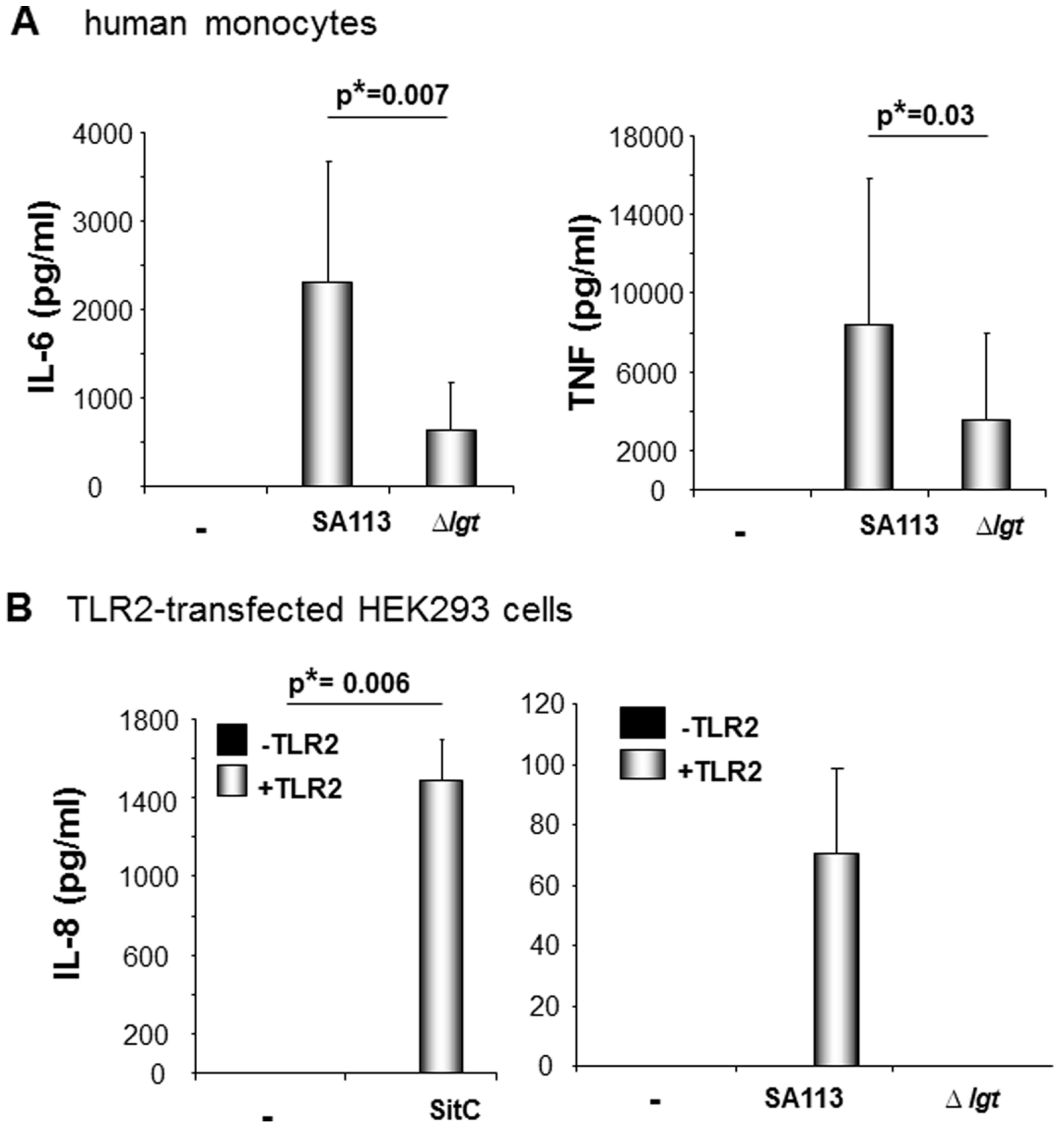
TLR2 ligands enhance *S. aureus*-induced cytokine secretion. **A:** Adherent human monocytes were stimulated with *S. aureus* strains: e.g. SA113 and its isogenic mutant Δ*lgt*, which is deficient in lipoprotein maturation. Secreted IL-6 (left) and TNF (right) concentrations were quantified in the supernatants. The diagrams show the mean values ± SD from cells from six healthy donors. **B:** HEK293 cells transfected with or without TLR2 plasmid (100 ng/well) were stimulated with *S. aureus* Lpp SitC (left; 5 ng/ml) or *S. aureus* strain SA113 (right). (**−**) refers to unstimulated cells. IL-8 was detected in the supernatants after 24h of stimulation. All diagrams show the mean values ± SD obtained in three experiments.

To date, the adjuvant potential of TLR2 ligands and their role in initiating and enhancing inflammation are well described. Illustrating this, the presence of TLR2-active lipoproteins (Lpp) enhances *S. aureus*-induced secretion of the proinflammatory cytokines IL-6 and TNF in human monocytes (Fig. 1A). However, bacterial immune stimulation does not depend on TLR2-activity, e.g. cytokine secretion remains detectable when cells are stimulated with a *S. aureus* strain lacking expression of TLR2-active lipoproteins (SA113 Δ*lgt*). Nevertheless, down-regulation of TLR2-activity is associated with reduced immune stimulation and might, thus, contribute to immune evasion.

To differentiate TLR2-activity from other immunostimulatory signals and to quantify and compare the extent of TLR2-activity among different clinical isolates we required a simple experimental system. As demonstrated in Fig. 1B HEK293 cells are not responsive to stimulation with the TLR2-active staphylococcal lipoprotein SitC or TLR2-active Lpp-expressing *S. aureus* strain SA113, unless transfected with TLR2 cDNA. Loss of TLR2-induced IL-8 secretion when using a staphylococcal mutants deficient in maturation of Lpp (*S. aureus* SA113 Δ*lgt*) confirmed the validity of our experimental system.

### Differences in Host TLR2 Recognition Among Clinical *S. aureus* Isolates

**Figure 2:**
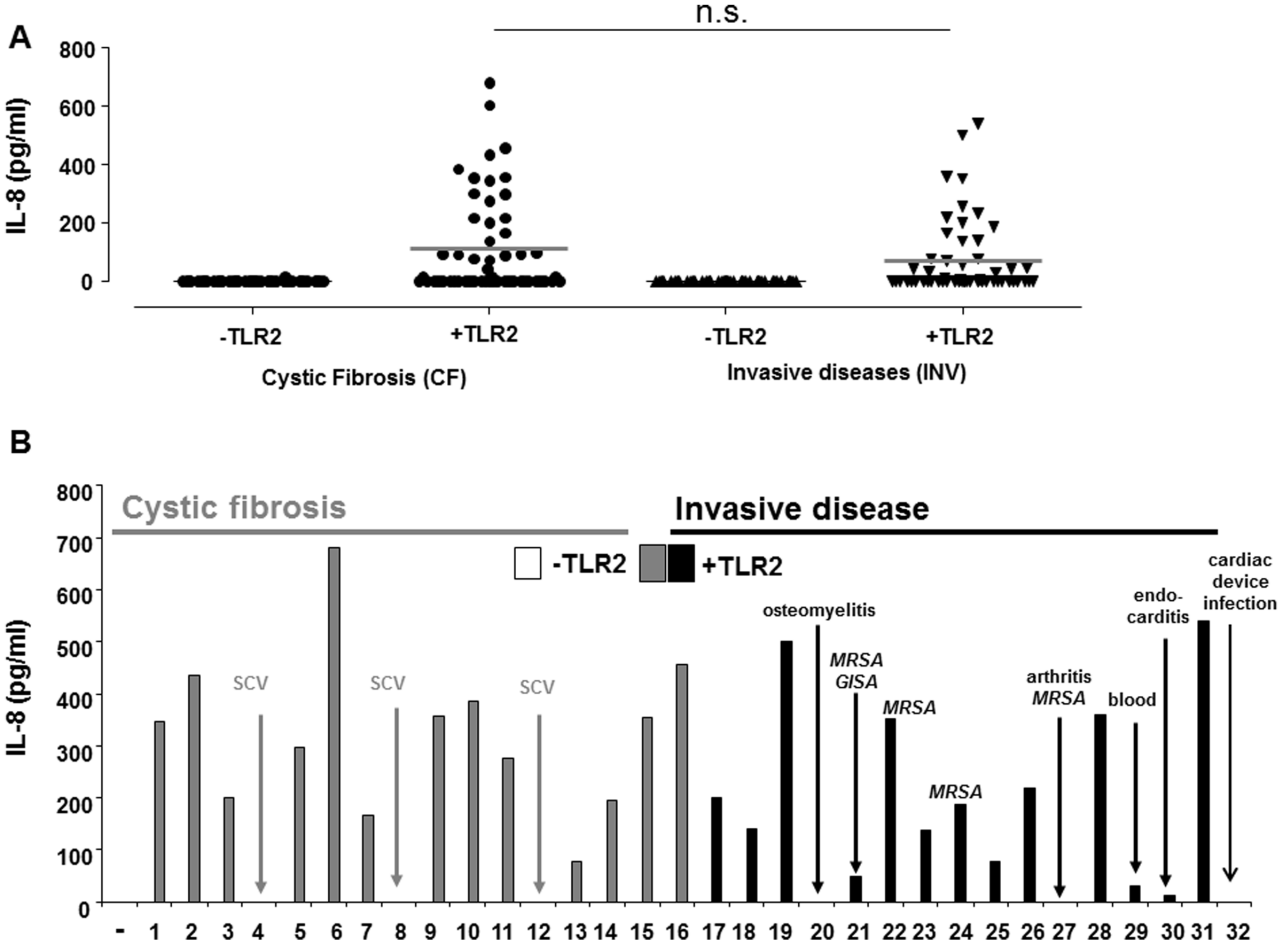
The TLR2-stimulatory capacity varies among *S. aureus* isolates. HEK293 cells transfected with or without TLR2 plasmid (100 ng/well) were stimulated with either cystic fibrosis (CF) or invasive (INV) *S. aureus* isolates. IL-8 production was quantified 24 hours after stimulation. **A:** TLR2-activity was tested in 53 CF (left) and 53 INV (right) isolates. The dots provide the values obtained for single strains, the bars indicate the means. No significant difference was observed when comparing CF to INV isolates. **B:** The diagram shows one representative experiment with 16 CF (left, grey) and 16 INV (right, black) isolates. The origins of the isolates with absent to low TLR2-activity (arrows) are indicated in the graph. (**−**) refers to unstimulated cells.

To assess whether intra-species variation also affects TLR2-activity we compared a 106 clinical *S. aureus* isolates with regard to their potential to induce TLR2-dependent IL-8 production in HEK293 cells (Fig. 2). The isolates used were taken from the clinical routine laboratory. One half of the isolates was derived from respiratory specimen from patients with cystic fibrosis (CF) because these isolates are of “colonizing nature”, e.g. this disease is associated with long-term *S. aureus* colonization of the respiratory tract; the other half of the strains were cultured from clinical specimen from patients with invasive diseases such as deep wound infection, meningitis or sepsis. The data obtained show that the *S. aureus* isolates display high variation in TLR2-stimulatory activity, e.g. strains with low (<60 pg/ml) to absent (64%; 68/106) and others with high TLR2-activity (≥60 pg/ml; 36%; 38/106) (Fig. 2). Notably, no significant differences were observed between the isolates from CF specimen, which predominantly comprise “colonizing isolates” and those cultured from overt infections, e.g. “invasive pathogens” (Fig. 2A). A more detailed analysis revealed that some of the S. *aureus* isolates lacking host TLR2-activity were small colony variants (SCV) (Fig. 2B, left panel), others were obtained from chronic infections such as osteomyelitis or endocarditis (Fig. 2B, right panel). Nevertheless, albeit reduced TLR2-activity was overrepresented in chronic infection this trend could not be confirmed by statistical analysis.

### Capsular Polysaccharides Mask TLR2 Active Lipoproteins

**Figure 3:**
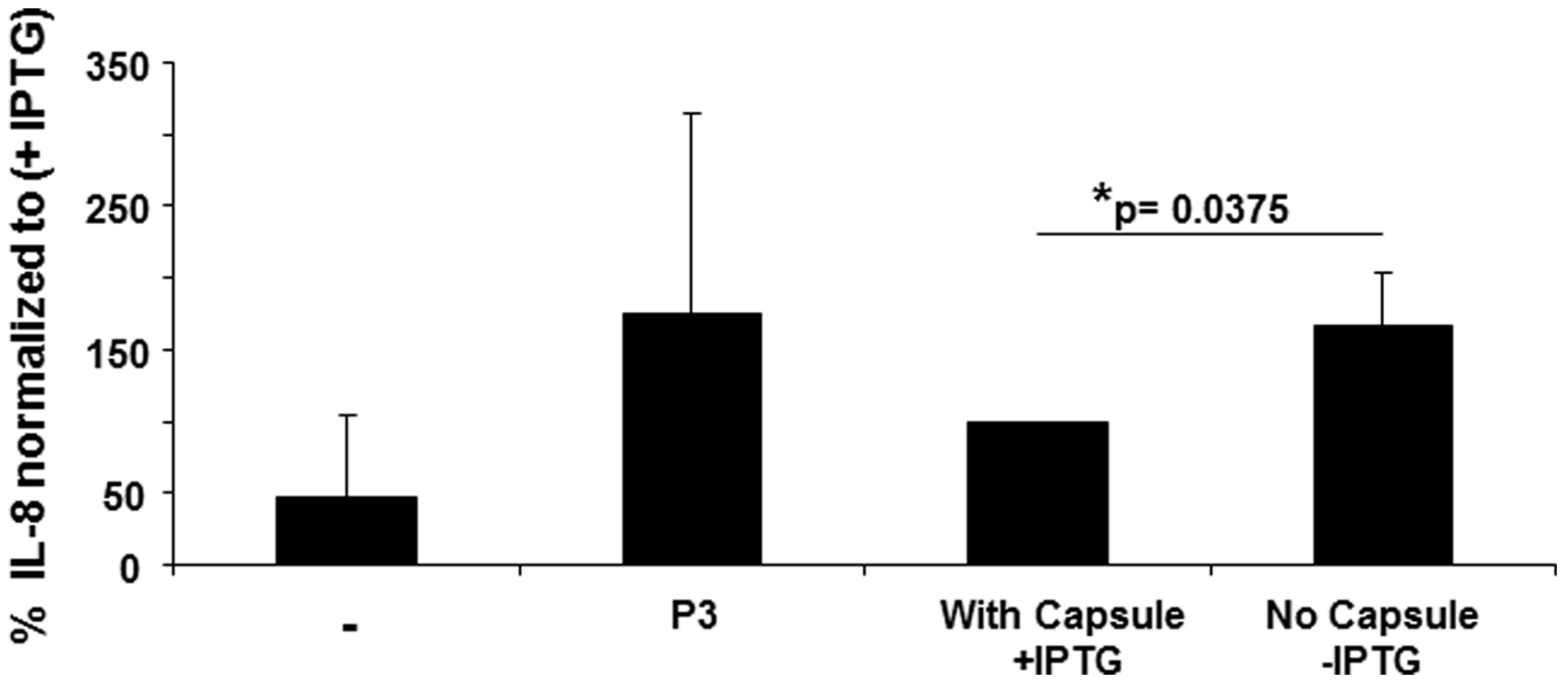
Capsular polysaccharides mask TLR2-activity in *S. aureus*. **A:** HEK293 cells were transfected with pTLR2 (200 ng/well) and stimulated with 1×10^6^ CFU/well of *S. aureus* Newman-132 mutant strain after culture with or without IPTG. Stimulation with bacteria was performed for 2.5 hours in the absence of antibiotics. Thereafter cells were washed and medium exchanged by RPMI medium containing ciprofloxacin to kill any residual bacteria. Cellular supernatants were harvested after 24 hours and analyzed for IL-8 concentrations. (**−**) refers to unstimulated cells, P3: Pam_3_CSK_4_ was used as positive control (200 ng/ml). Results were normalized to Newman-132+IPTG ( = 160.4 pg/ml ±121.6) = 100%; Newman-132 -IPTG = 292.4 pg/ml ±268.5. The diagram shows the results obtained in four experiments given as mean values ± SD.

It was previously reported that the loss of capsular polysaccharides expression in staphylococci enhances the interaction between bacterial surface proteins and host cell receptors [18], [19]. We reasoned that capsule expression could also interfere with Lpp recognition by TLR2. To investigate this finding we used a *S. aureus* Newman mutant (Newman-132), with an IPTG (isopropyl-β-D-1 thio-galacto-pyranoside)-inducible *cap5* promoter [13]. To determine the differences in TLR2-triggered IL-8 secretion levels with and without induction of capsular polysaccharide expression we had to refine the experimental conditions: bacteria were grown in liquid cultures with and without IPTG supplement for 6 hours; equivalent amounts of bacteria were added to HEK293 cells in the absence of antibiotic supplement; bacterial stimulation was terminated after 2.5 hours to avoid stimulatory effects from cell decay; following culture medium exchange and addition of ciprofloxacin HEK293 cell supernatants were collected after 18 hours. Indeed, encapsulated Newman-132 (+IPTG) displayed less stimulatory activity for host TLR2 than the unencapsulated strain (Fig. 3). This indicated that expression of capsular polysaccharides interferes with lipoprotein recognition and TLR2 stimulation.

### Protein A Expression Correlates with Strain-specific Levels of Host TLR2-activity

**Figure 4:**
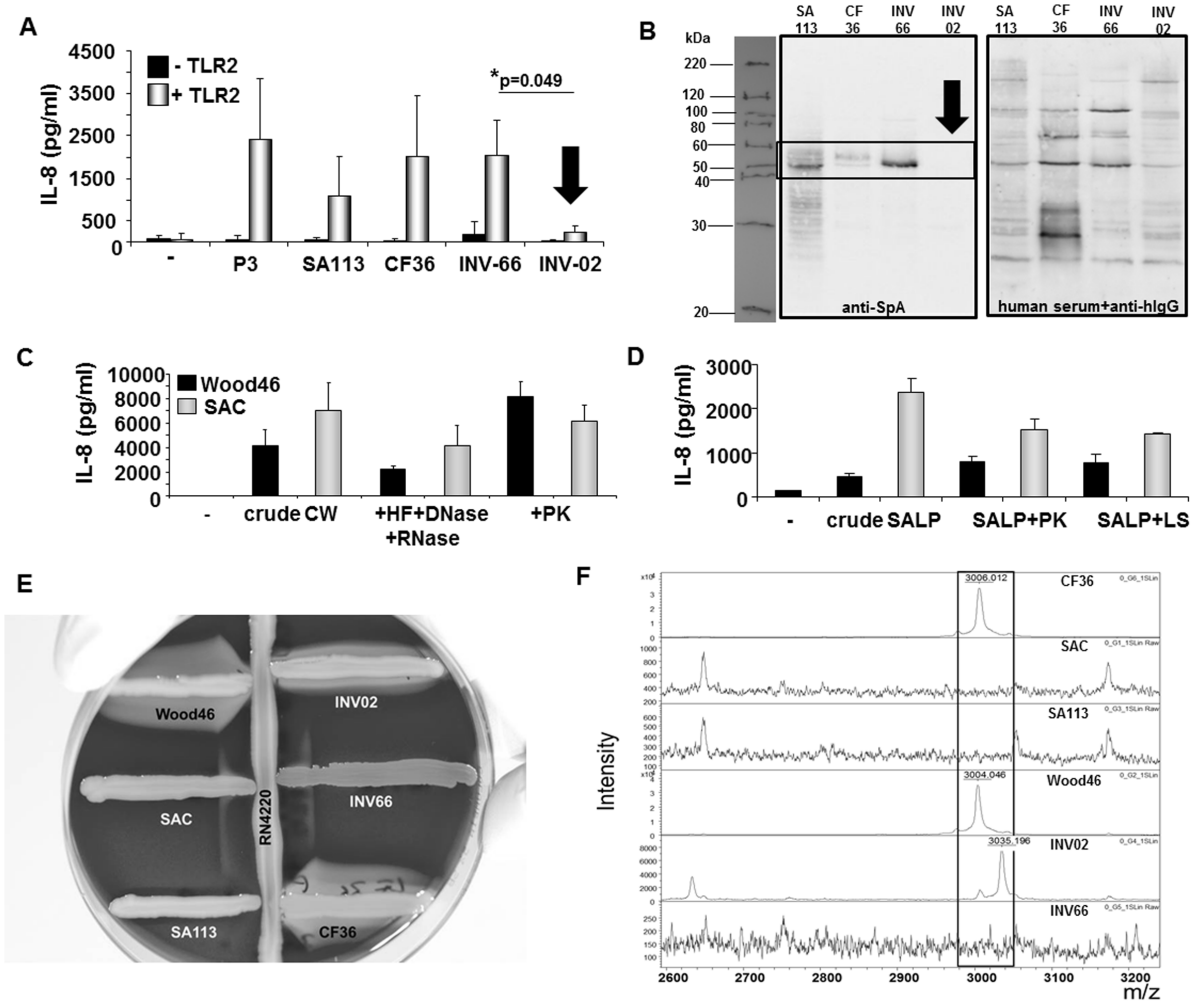
Protein A expression correlates with TLR2-activity. **A:** Comparison of TLR2-induced IL-8 secretion levels after stimulation of HEK293 cells transfected with 200 ng/well of pTLR2 with SA113, clinical *S. aureus* isolates (CF36, INV66, INV02), 1 µg/ml Pam_3_CSK_4_ (P3) or when left unstimulated (**−**). **B:** Comparison of SpA expression in SA113 and clinical *S. aureus* isolates (CF36, INV66, INV02). Left: Western blot analysis of SpA expression in bacterial lysates using anti-SpA mAb and anti-murine IgG-HRP. Right: Control blot incubated with human serum and biotinylated anti-human IgG-+ streptavidin-HRP to visualize protein loading. One representative experiment of n ≥ 3 experiments is shown. **C+D:** Stimulation of TLR2-transfected HEK293 cells with 5 µg/ml cell wall (CW; **C**) or lipoprotein preparations (SALP; **D**) prepared from *S. aureus* strains Cowan I (SAC; SpA^high^, grey bars) or Wood46 (SpA^low^, black bars). As indicated crude CW (left) were treated with hydrofluoric acid (HF) and RNAse A and DNAse I (middle) followed by digestion with proteinase K (PK, right). SALP were treated with proteinase K (PK, left) or lysostaphin (LS, right). (**−**) refers to unstimulated cells. The experiments shown were performed in triplicates and show mean values ± SEM of IL-8 concentrations determined in the supernatants. They are representative of n = 2 independent experiments. **E+F:** **Analysis of** ***agr*** **activity. E: Hemolysin production.** Strains to be tested for hemolysin production were cross-streaked to RN4220, which produces β-hemolysin. After incubation for 36 hours strains were analyzed following the description by Taber et al. [15]: Wood46 and CF36 displayed the typical pattern for α- and δ-hemolysin expression, INV02 produced α-, β- and δ-hemolysins, SAC (Cowan I) and INV66 expressed only low amounts of δ-hemolysin and SA113 was negative for α-, β- and δ-hemolysins. In conclusion, SA113, SAC and INV66 were categorized as *agr*-, Wood46, CF36 and INV02 were typed as *agr*+. **F: Analysis of δ-toxin expression by MALDI-TOF.** δ-toxin expression is dependent on *agr* activity as reported in [17]. Delta toxin (MW 3007) and delta toxin G10S (MW 3037) peaks were detectable in Wood46, INV002 and CF36 and absent in SAC, SA113 and INV66.

We previously described a correlation of protein A (SpA) expression and TLR2-activity in SCV and reduced TLR2-activity in *spa*-deficient SA113 [14]. This correlation was confirmed in the clinical isolates used for the present study: strains with high TLR2-activity expressed high levels of SpA (i.e. INV66), while SpA expression was low to absent in strains with low to absent TLR2-activity (i.e. INV02) (Fig. 4A+B).

SpA expression is high during the exponential phase of growth. To exclude the effects of bacterial proliferation on TLR2-activity, we, next, compared TLR2-activity in crude cell wall preparations and lipoprotein fractions from SpA^high^ Cowan strain I (SAC) and SpA^low^ Wood46 [20]. The results revealed that preparations from both strains induced IL-8 in TLR2-expressing HEK293 cells (Fig. 4C+D). Notably, crude preparations derived from SAC were better stimulators of TLR2-induced IL-8 than those from Wood46. However, strain-dependent differences in TLR2-activity became negligible after digestion of cell walls or lipoprotein fractions with proteinase K or lysostaphin (Fig. 4C+D), which results in elimination of SpA (see [12]).

In the post-exponential growth phase *agr* is turned on and promotes toxin secretion and down-regulation of the synthesis of cell wall-anchored proteins such as SpA. To exclude *agr* as a regulator of host TLR2-activity we assessed *agr* activity based on presence of δ-haemolysin production as described in [15] and [21]. Testing of representative strains for δ-hemolysin expression on sheep red blood agar plates (Fig. 4E) and by MALDI-TOF mass spectrum analysis (Fig. 4F) revealed no overt correlation of *agr* activity with TLR2-induced IL-8 secretion in HEK293 cells. For example, clinical isolates CF36 (SpA^intermed^, TLR2/IL-8^high^) and INV02 (SpA^low^, TLR2/IL-8^low^) were identified as *agr* positive, while clinical isolate INV66 (SpA^high^, TLR2/IL-8^high^) and SA113 (SpA^high^, TLR2/IL-8^high^) were *agr* negative. Furthermore, Wood46 (*agr+,*SpA^low^) and SAC (*agr−,* SpA^high^) were both TLR2/IL-8^high^ (data not shown). Furthermore, comparison of host TLR2-activity in the Newman strain with an isogenic Newman Δ*agr* mutant revealed no significant difference in TLR2-activity in our experimental context (data not shown). Taken together, these experiments indicated that TLR2-activity is independent of the *agr* status and but enhanced in the presence of SpA.

### Small Colony Variant Formation can be Associated with Reduced TLR2-activity

The SCV phenotype is associated with slow growth and deficits in cell metabolism together with intracellular persistence (reviewed in ([22], [23]. SCV are most frequently encountered in CF patients and chronic infections because formation is favored by long-term antibiotic treatment [22], [24]. In views of our observation that some of the isolates with nearly absent TLR2-activity were SCV, we asked whether SCV formation is generally associated with lower TLR2-activity. We, therefore, sought to investigate whether the SCV phenotype is associated with a loss in host TLR2 recognition when compared to normal isolates.

**Figure 5:**
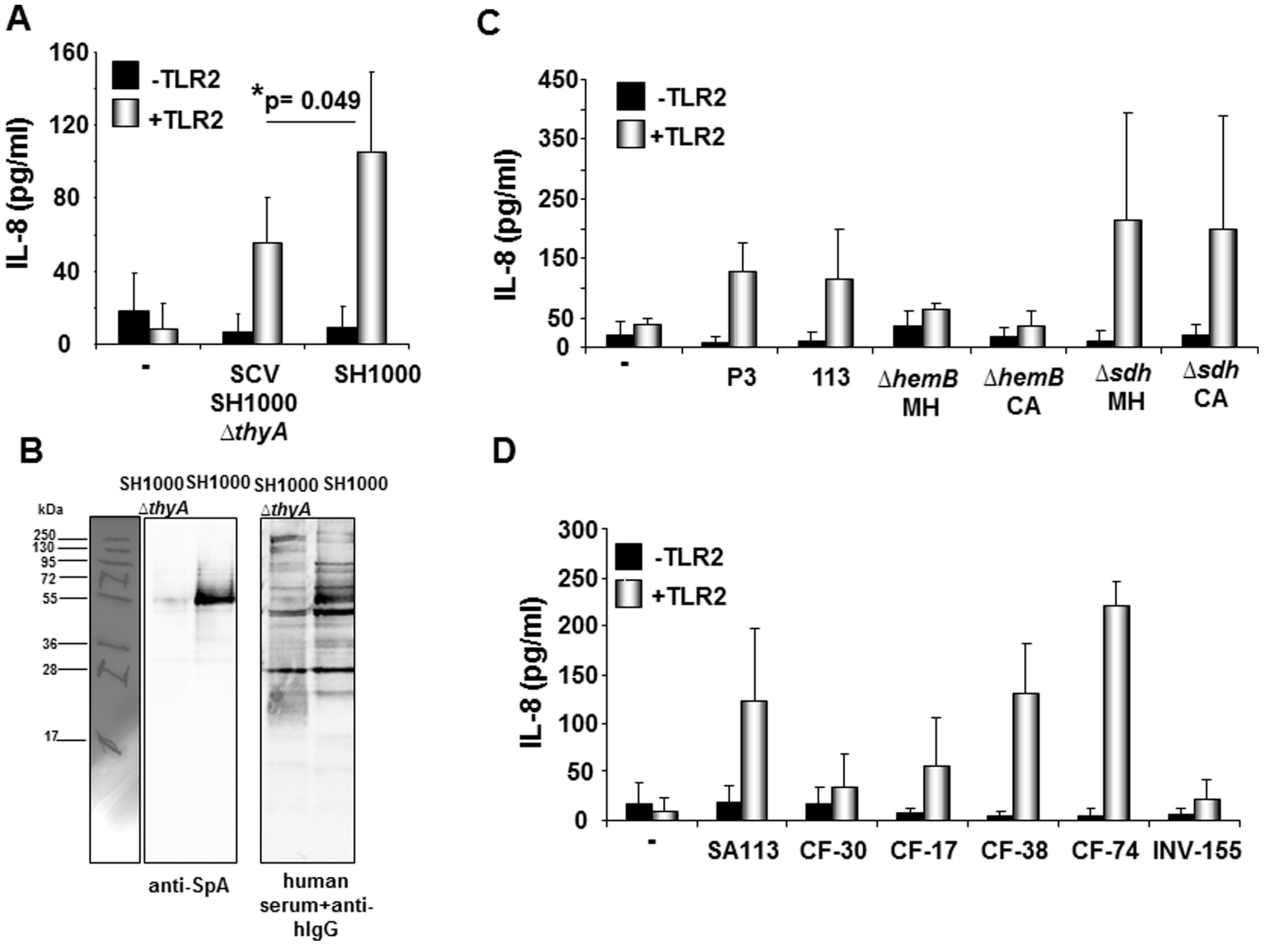
SCV formation can be associated with reduced TLR2-activity. **A,C and D:** HEK293 cells were transfected with or without TLR2 cDNA (100 ng/well in **A+D** or 200 ng/well in **C**) and stimulated with *S. aureus* strains for 18–20 hours. IL-8 secretion was quantified as indicator of TLR2 activation. All diagrams show the results obtained in three independent experiments given as mean values ± SD (standard deviation). **A:** Stimulation of HEK293 cells w/o pTLR2 with *S. aureus* SH1000 and its isogenic thymidine-auxotrophic mutant SCV SH1000 *ΔthyA*; *p(SH1000:*ΔthyA) = *0.049. **B:** Comparison of SpA expression in SH1000 (TLR2^high^) and its isogenic SCV SH1000 *ΔthyA* (TLR2^low^). Left: Western blot analysis of SpA expression in bacterial lysates using anti-SpA mAb and anti-murine IgG-HRP as secondary antibody. Right: Control blot incubated with human serum and biotinylated anti-human IgG+streptavidin-HRP. One representative experiment of n = 3 experiment is shown. **C:** HEK293 cells w/o TLR2 were stimulated with SA113 or its mutants *ΔhemB* and Δ*sdh* (grown on Mueller Hinton (MH) or Columbia agar (CA)) or with 1 µg/ml of the TLR2 ligand Pam_3_CSK_4_ (P3) or left unstimulated (**−**). **D:** Stimulation with five different clinical *S. aureus* SCV (isolate source: CF = cystic fibrosis, INV = invasive) and SA113. (**−**) = unstimulated.

To investigate whether loss of host TLR2 stimulatory potential is a characteristic of SCV we, first, retrieved to genetically defined mutants that result in SCV formation, but remain genetically comparable to their wild type counterparts. Indeed, TLR2-activity determined in *S. aureus* SH1000 *ΔthyA* was lower than that in the isogenic SH1000 strain (Fig. 5A) [25], [26]. Well in line with this finding, the SH1000 Δ*thyA* mutant expressed reduced levels of SpA (Fig. 5B). Next, we observed that compared to the SA113 parental strain TLR2-dependent IL-8 induction was reduced in the SA113 *ΔhemB* mutant and increased in the SCV-like SA113 Δ*sdh* mutant (Fig. 5C).

Confirming these data, comparison of 5 clinical *S. aureus* isolates with SCV phenotypes revealed that these strains displayed varying levels of host TLR2-activity (Fig. 5D): low IL-8 levels (<60 pg/ml) in CF30, CF17 and INV155 and elevated levels (≥60 pg/ml) in CF38 and CF74. In conclusion, SCV formation can be associated with a reduction in TLR2-activity but this is not an obligatory finding.

### Disruption of Cell Wall Integrity Facilitates Sensing of TLR2 Ligands

TLR2-active lipoproteins (Lpp) are localized at the cytoplasmic membrane of Gram positive bacteria, including *S. aureus* [27], [28]. Lpp biosynthesis involves Lpp acylation and sorting to the cell wall. If any of these steps is defective staphylococci lose TLR2-activity [27]. Loss of TLR2-activity in clinical isolates could, thus, be caused by defective Lpp biosynthesis. We reasoned that disruption of cell wall integrity could help us to differentiate between total lack of di- and tri-acylated (TLR2-active) Lpp (defective Lpp maturation) and loss of TLR2-activity due to changes in the cell wall architecture or defective sorting.

**Figure 6:**
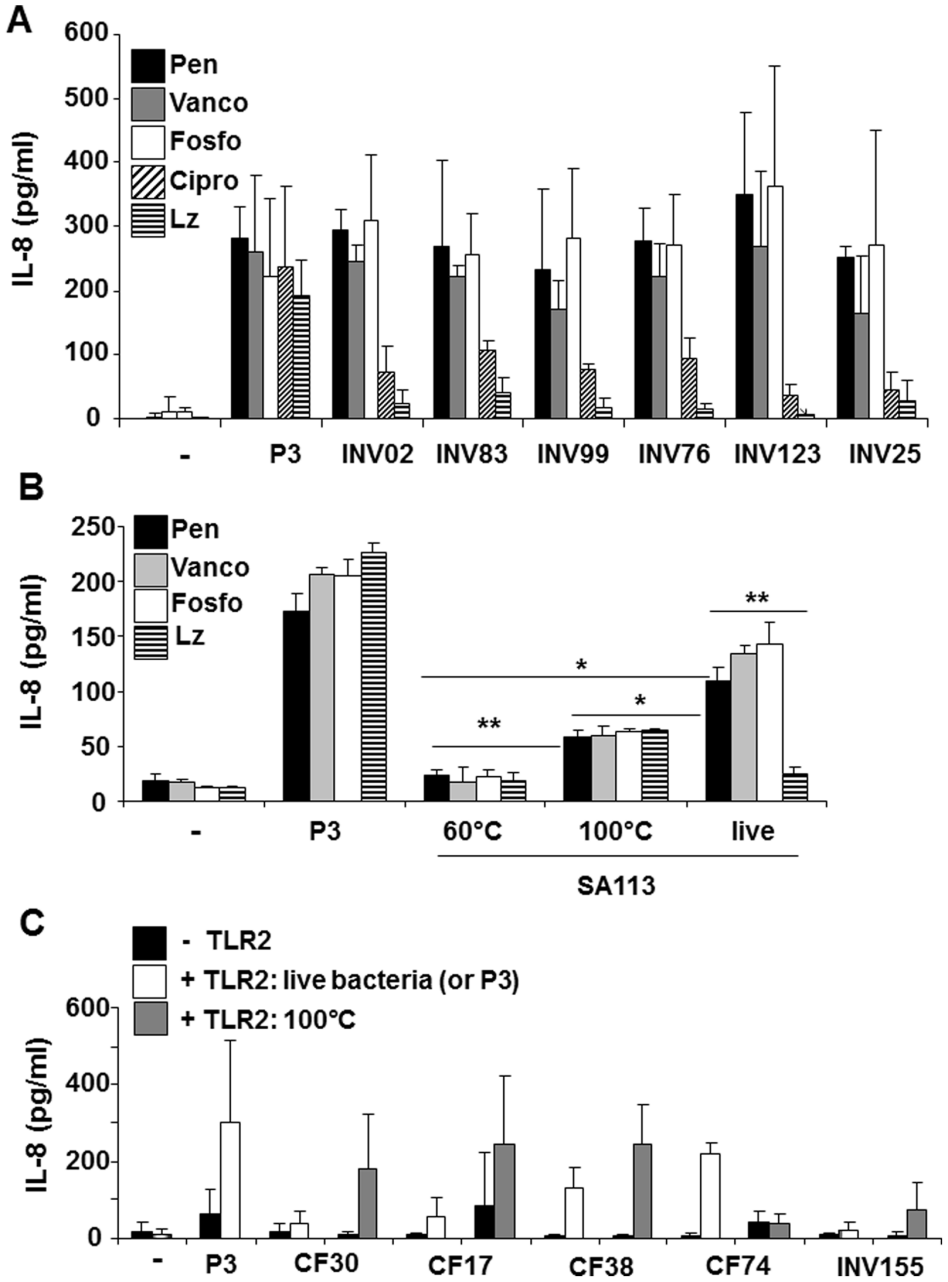
Disruption of cell wall integrity facilitates recognition of TLR2 ligands. **A+B:** HEK293 cells transfected with 100 ng/well of TLR2 plasmid were stimulated with clinical *S. aureus* isolates (**A**) or with heat-inactivated, boiled or live SA113 (**B**) and compared to Pam_3_CSK_4_ (P3; 0.2 µg/ml (**A**); 1 µg/ml (**B**)) and to unstimulated cells (**−**). Stimulation was carried out in the presence of different antibiotics: penicillin (Pen), vancomycin (Vanco), fosfomycin (Fosfo), ciprofloxacin (Cipro) or linezolid (Lz). *S. aureus* isolates were susceptible to all antibiotics tested. The diagrams shown provide the mean values ± SD of IL-8 values summarized from n = 3 independent experiments. The most relevant statistical results are highlighted as (*) or (**): Penicillin: *p (live:100°C) = 0.03, *p (live:60°C) = 0.01, **p (100°C: 60°C) = 0.001 and **p (live+Pen: live+Lz) = 0.002; not indicated: Vancomycin: **p (live:100°C) = 0.001; Linezolid: *p(live:100°C) = 0.006. **C:** HEK293 cells transfected with (white or grey bars) or without (black bars) 100 ng/well of TLR2 plasmid were stimulated with clinical SCV strains, 0.2 µg/ml of Pam_3_CSK_4_ (P3) or left unstimulated**.** Live bacteria (white bars) were compared to crude preps (boiled bacterial suspensions; grey bars). IL-8 levels in the supernatants were determined to quantify TLR2-activity. The diagram shows the mean values ± SD obtained in three independent experiments.

To tackle this issue we, first, used antibiotics to interfere with cell wall integrity. Treatment of susceptible bacteria with cell wall synthesis inhibitors, e.g. penicillin, vancomycin or fosfomycin, supported IL-8 induction in TLR2-transfected HEK293 cells. By contrast, TLR2-activity remained low after exposure of bacteria to antibiotics that target nucleic acid and protein synthesis, i.e. ciprofloxacin, which targets the DNA gyrase, or linezolid, a protein synthesis inhibitor (Fig. 6A). Notably, activity of synthetic TLR2 ligand Pam_3_CSK_4_ was not influenced by the addition of antibiotics (Fig. 6A). These experiments allowed two conclusions: firstly, TLR2 stimulation is detectable in spite of inhibition of core cell functions such as protein synthesis and, secondly, breaking up the cell wall enhances TLR2-mediated IL-8 production.

Of note, inactivation of bacteria at 60°C, which preserves the cell wall structure but prevents growth and metabolic activity, reduces host TLR2-activity to nearly the background levels measured in unstimulated HEK293 cells or in the presence of linezolid (Fig. 6B). By contrast, boiling of cells at 100°C, which denatures proteins and nucleic acids, but preserves lipids and TLR2-activity, resulted in IL-8 levels above those induced by heat-inactivated *S. aureus* (Fig. 6B). Disruption by boiling, thus, increases host TLR2 recognition when compared to intact cells.

As seen with Pam_3_CSK_4_ antibiotic treatment did not affect TLR2-activity in bacteria treated at 60°C or 100°C (Fig. 6B). Bacterial viability was, however, a prerequisite for the increase in host TLR2 activation observed after treatment with cell wall-targeting antibiotics. Notably, TLR2-activity measured after antibiotic-mediated interference with peptidoglycan crosslinking was higher than that obtained with crude (“boiled”) cell preparations. This indicated that release of peptidoglycan fragments during ongoing cell wall synthesis provide an additional increase in TLR2 stimulation. Altogether, our data showed that disruption of cell wall integrity as achieved by boiling or cell wall-targeting antibiotics facilitates Lpp recognition by TLR2.

### TLR2-activity is Preserved in Strains Expressing PBP2a

Methicillin-resistant *S. aureus* (MRSA) carries the alternate penicillin-binding protein PBP2a. In views of the results shown in Fig. 6A+B we asked whether PBP2a expression or resistance to betalactam antibiotics influenced our assessment of TLR2-activity. As expected, experiments with MRSA carried out in the presence of penicillin only were inutile due to bacterial overgrowth (data not shown). However, in the presence of streptomycin (Fig. 2B) or vancomycin (data not shown) we were able to assess TLR2-activatory potentials: similarly to methicillin-sensitive isolates (MSSA), strains displayed low TLR2-activity, i.e. INV21 and INV27 (Fig. 2B) or SCV INV155 (Fig.5D) or high TLR2-activity, i.e. INV22 and INV24 (Fig.2B). These data corroborated that TLR2-activity in MRSA isolates is strain-dependent and differences are not blunted by betalactam resistance.

### TLR2^low^ SCV Contain TLR2-active Lipoproteins

To better understand the reasons for low TLR2 stimulatory capacity in clinical SCV isolates we prepared crude bacterial preparations (100°C). Notably, boiling restored TLR2-triggered IL-8 secretion in all but one of the crude preparations obtained from TLR2/IL-8^low^ SCV tested (Fig. 6C). This indicated that all SCV strains contain mature Lpp and that Lpp accessibility represents the limiting factor. SCV-typical changes in cell wall structure and permeability could account for reduced TLR2 activation.

## Discussion

Recognition of *Staphylococcus aureus* via TLR2 can be controlled by both host and pathogen. In this study we challenge the current view that high TLR2 stimulatory capacity is an intrinsic property of the staphylococcal species. Based on comparison of clinical isolates we provide evidence that the ability to trigger TLR2 in the TLR2-transfected HEK293 cell system varies on a strain-to-strain-dependent basis (Fig. 2). Thus, the genetic heterogeneity typical of *S. aureus* translates into differences in TLR2 activating potential.

We further analyzed possible mechanisms that control bacterial TLR2-activity and might modulate Lpp recognition *in vivo.* It was previously demonstrated that capsule expression interferes with pathogen-host cell interaction [18], [19]. Our findings indicate that expression of capsular polysaccharides, common in clinical isolates [29], [30], interferes with bacterial recognition via TLR2 (Fig. 3). Masking of TLR2-active Lpp by capsular polysaccharides could result in ineffective immune recognition and, thus, facilitate invasive infection.

Interestingly, capsule expression is often lost during chronic infection [31]. This raises the question why this could represent an advantage for the pathogen. In views of the fact that many SCV isolates lose their TLR2 activatory potential (Fig. 2 and 5) and that other isolates with only low to absent induction of host TLR2-activity were cultured from chronic infections such as endocarditis (INV30), osteomyelitis (INV20) or deep wound infections after implant of cardiac devices (INV32) (Fig. 2), it is well conceivable that loss of TLR2 activation capacity accompanies host adaptation and reduces the need for capsule expression. Loss of both capsule and TLR2-active Lpp makes the bacteria less visible for the immune system.

Special attention should be given to reduced TLR2 stimulatory potential in SCV isolates (Fig. 5). The analyses showed that the majority of SCVs tested in this study triggered only low TLR2-dependent IL-8 secretion levels (Fig. 5D). The experiments conducted with genetically targeted mutants forming SCV also indicated that SCV formation is frequently associated with loss of TLR2-activity (Fig. 5A+C). Little is known on the regulation of Lpp expression in *S. aureus*. Lower TLR2 stimulation with SCV could be due to a reduction in protein synthesis and turnover associated with the SCV phenotype. Similarly, inhibition of protein synthesis with linezolid lowered TLR2-induced IL-8 levels (Fig. 6A+B). TLR2 antagonizing properties of SCV could further include down-regulation of cell wall synthesis and defective Lpp sorting because cell disruption by boiling restores or enhances TLR2 recognition in all but one SCV isolate (Fig. 6C).

Notably, increased TLR2 responsiveness in the presence of cell wall-targeting antibiotics was not observed in non-dividing heat-treated cells (Fig. 6B). However, when comparing live bacteria differences in proliferation and cell division might influence differences in TLR2-activity and the presence of cell wall synthesis inhibitors could accentuate these. Slow growth as seen with SCV and in chronic infections might, in turn, dampen TLR2 recognition. Furthermore, ongoing cell proliferation is accompanied by release of peptidoglycan fragments, accumulation of dead cells and increased cell wall permeability, which increases the extent of TLR2 activation by facilitating access of TLR2 to Lpp.

In the drosophila it has previously been demonstrated that the recognition of extracellular bacteria by the host cell is negatively regulated by surface receptors with amidase function that inactivate peptidoglycan by cleavage [32]. These receptors prevent peptidoglycan sensing unless concentrations exceed the threshold that enables cellular activation, usually achieved in the presence of proliferating bacteria. In views of the fact that in our experimental system the extent of host TLR2 activation most likely depends on peptidoglycan and Lpp release associated with proliferation or use of cell wall-targeting antibiotics, we propose that under *in vivo* conditions TLR2 serves as an indicator for “free” Lpp. In support of this hypothesis Moore et al. described that treatment of *S. pneumoniae* with penicillin releases bacterial cell-wall components that activate the proinflammatory response via TLR2, a mechanism mediated by the autolytic enzyme LytA [33].

Nevertheless, the experiments carried out in the absence of antibiotics (Fig. 3), with heat-treated bacteria (Fig. 6B) or cell wall and Lpp preparations (Fig. 4C+D) indicate that host TLR2 recognition does not solely depend on bacterial metabolism and proliferation. In a previous report we made the observation that the degree of TLR2-stimulatory activity in a series of SCV isolates correlated with SpA expression and was reduced in the *spa*-deficient SA113 mutant [14]. Albeit the mechanism remains to be investigated, we corroborated these findings in present study: under the growth conditions used in this study the comparison of SCV SH1000 Δ*thyA* mutant and the SH1000 strain revealed reduced expression of SpA and loss of TLR2-activity in the SCV (Fig. 4B); differences in TLR2-activity in cell wall and Lpp preparations of SpA^high^ (SAC) and SpA^low^ (Wood46) strains were revoked after depletion of SpA with proteinase K or lysostaphin treatment (Fig. 4C+D); lastly, a correlation of SpA levels with strain-dependent TLR2 recognition was observed in clinical isolates (represented by INV02, INV66 and CF36 in Fig. 4A+B), but a role for *agr* was excluded (Fig. 4E+F).

Nevertheless, it needs to be emphasized that despite their adjuvant properties TLR2-active Lpp alone do not account for the total immune stimulatory capacity of a single *S. aureus* strain (Fig. 1). Recognition of TLR2-active Lpp represents only one out of several bacterial components whose presence or absence shapes the immune response to *S. aureus*. Taken together our findings imply that TLR2-activity is influenced by multiple factors, which include proliferative activity, capsule formation, protein synthesis and cell wall-intrinsic factors such as quantitative expression of Lpp or SpA. We can only speculate that in chronic infections down-regulation of cell wall and protein synthesis contributes to the evasion of TLR2 recognition and subsequently facilitates host adaptation and persistence.
